# Incidence and predictors of loss to follow-up among HIV infected adults on antiretroviral therapy in Guji Zone, Southern Ethiopia, 2023

**DOI:** 10.1186/s12879-026-13965-5

**Published:** 2026-07-09

**Authors:** Dereje Girma, Gemechis Tuke, Zelalem Jebessa, Kebebew Lemma, Azmach Habtegiorgis, Ashenafi Lemesa, Alo Edin

**Affiliations:** 1 Oromia Health Bureau, Disease Prevention and Control Department, Shakiso Town Health Office, Shakiso, Ethiopia; 2 Department of Public Health, Institute of Health, Bule Hora University, Bule Hora, Ethiopia; 3 Department of Nursing, Institute of Health, Bule Hora University, Bule Hora, Ethiopia

**Keywords:** HIV, Antiretroviral therapy, Loss to follow up, Incidence, Risk factors, Ethiopia

## Abstract

**Background:**

Antiretroviral therapy (ART) has significantly improved outcomes for people living with HIV (PLHIV) globally. However, loss to follow-up (LTFU) remains a major barrier to the effectiveness of ART programs, particularly in resource-limited settings. In Ethiopia, LTFU contributes substantially to patient attrition, yet data from high-burden zones like Guji remain limited. Therefore, this study aimed to determine the incidence rate and identify predictors of loss to follow-up among HIV-infected adults receiving ART at public health facilities in Guji zone, Southern Ethiopia, 2023.

**Methods:**

An institution-based retrospective cohort study was conducted among 434 HIV-positive adults who initiated ART between July 2018 and June 2022 at public health facilities in Guji zone. Participants were selected using simple random sampling. Data were extracted from medical records using a structured checklist and entered into Epi Data 4.6.2. Analysis was performed using SPSS 25 and STATA 14. The incidence rate of LTFU (defined as >30 days since last scheduled appointment date and has not been classified as “dead” or “transferring out) was calculated per 100 person-years. Bivariable and multivariable Cox proportional hazards regression were used to identify predictors of LTFU. Adjusted hazard ratios (AHR) with 95% confidence intervals and *p*-values < 0.05 were considered statistically significant.

**Result:**

Among 434 participants (60% female) followed for 904.35 person-years, 135 (31.1%) were lost to follow-up, yielding an incidence rate of 14.93 per 100 person-years. Factors significantly associated with higher LTFU risk were: male sex (AHR = 1.85, 95%CI: 1.27–2.69), age 15–30 years (AHR = 2.40, 95%CI: 1.58–3.64), female sex work (AHR = 3.26, 95%CI: 1.39–7.65), daily labor/mobile work (AHR = 1.91, 95%CI: 1.16–3.16), and no formal education (AHR = 2.20, 95%CI: 1.52–3.20). Protective factors associated with lower LTFU risk were: residing in the same woreda as the health facility (AHR = 0.55, 95%CI: 0.38–0.82) and having a documented phone number (AHR = 0.46, 95%CI: 0.31–0.67).

**Conclusion:**

The incidence of LTFU among adults on ART in Guji zone was high, particularly during the first year of treatment. Male sex, younger age, lack of formal education, female sex work, and daily labor were associated with increased LTFU risk, while residing in the same woreda as the health facility and having documented phone numbers were protective. Targeted interventions including enhanced counseling for high-risk groups, community-based ART refill models, and active phone-based tracking systems should be implemented to improve retention.

## Background

Human Immunodeficiency Virus (HIV) is a chronic infectious disease characterized by a spectrum beginning with primary infection, with or without acute syndrome, followed by a lengthy period of asymptomatic stage, after which most patients progress to advanced and life-threatening disease called acquired immune deficiency syndrome (AIDS) [1, 2].

As of the end of 2021, there were 38.4 million HIV-positive people worldwide, up from 25.5 million in 2000 [3]. In Africa 25.78 million HIV-positive people were living as of the end of 2021 [4]. Eastern and Southern Africa continues to be the area most severely affected by the disease, accounting for 54% of all individuals living with HIV and two-thirds of all children living with HIV, despite a 44% decline in new infections from 2010 to 2021 [3, 4].

Ethiopia is one of the countries that has a long history of having a widespread HIV epidemic [5]. Anti-retroviral therapy (ART) was a great innovation that has helped to significantly lower HIV-related mortality, admissions, and poor outcomes such as transmission [6–8].

There are 617,921 persons living with HIV (PLWHA) in Ethiopia as of 2021 estimations and forecasts relating to HIV, with an anticipated adult HIV prevalence of 0.93% [7]. The invention of antiretroviral changed the treatment of HIV-infected patients and resulted in notable decreases in HIV-associated morbidity and mortality in many developed nations [6, 9, 10]. ART has demonstrated effectiveness in lowering mortality among patients who continue receiving treatment and following recommended treatments [3, 4, 6, 10]. During the period from 2010 to 2018, Ethiopia experienced a 45% decline in the number of AIDS-related deaths, from 20 000 to 11 000, according to the UNAIDS annual report. Despite declines in incidence of HIV and the advantages of greatly increased antiretroviral access, HIV transmission remains high and is associated with substantial morbidity and mortality [5–7]. Ethiopia did not achieve the UNAIDS 90–90-90 targets by 2020, with poor patient retention and high LTFU rates being major contributing factors [5, 8, 11, 12].

It is globally agreed to bring an end to HIV/AIDS new infection by 2030 [6, 13]. The Fast-Track approach is a global implementation strategy with a special focus on 30 countries that house 89% of all newly infected HIV patients worldwide [13]. As part of this strategy, UNAIDS set a 95–95–95 target, which states that 95% of persons with HIV will be aware of their status, 95% will be receiving treatment, and 95% will have viral levels that have been suppressed [13]. Ethiopia is one of the main targeted countries for UNAIDS’ Fast-Track strategy, which calls for increasing HIV care and treatment programs with higher standards [13].

Regular patient follow-up is essential for the ART program’s success. Hence, HIV-positive people must enroll in HIV care and adhere to their prescribed ART regimens without interruption to get the best results from their treatment [6, 13, 14]. Although multiple factors contribute to attrition among HIV-positive patients in Ethiopia, LTFU is the leading cause, followed by transfer out and death [15]. LTFU in ART has been identified as the main threat to the long-term success of these programs and is a barrier to its implementation in many sub-Saharan African nations [12, 14, 16]. The immunologic and viral benefits of ART are adversely affected by LTFU, which increases AIDS related morbidity, death, and hospitalizations [14, 17]. Additionally, it makes it more challenging to assess the efficacy of an HIV treatment program [14, 17].

Ethiopia faces significant challenges with patient adherence, as evidenced by multiple studies reporting high attrition and low retention rates [15, 18–20]. A 2020 systematic review of studies conducted across all regions of Ethiopia between 2005 and 2019 found an overall retention rate of 70.65%, with rates ranging from 94% in the first year of follow-up to 32.5% by the ninth year [15]. Another study conducted in Northwest Ethiopia among adult who started ART from January 1, 2008 to December 30, 2017 found the incidence rate of loss to follow up 13.45/100PY [21].

Studies revealed that LTFU from ART among adults was predicted by several sociodemographic and economic, behavioral, family support, facility-related, and clinical factors [15, 16, 22–26]. The Loss to follow-up (LTFU) problem persists, particularly in resource-limited settings and it can be generalized that LTFU is a significant challenge that prohibits antiretroviral therapy from being effective and raises morbidity and mortality(4,6,8,15,16,27. This can hinder ART program advancement and the global goal of ending AIDS as a public health threat by 2030 [3, 13].

Although several studies on loss to follow-up (LTFU) have been conducted in Ethiopia, there is limited published evidence specific to the Guji zone, an area with reportedly high rates of new HIV infections and low ART retention according to zonal health reports [27]. Furthermore, Guji Zone has unique contextual characteristics, including population mobility associated with mining activities and pastoralist livelihoods in some areas, where access to healthcare may be constrained by geographic remoteness and limited transportation options. These factors may influence retention in HIV care and limit the generalization of findings from other Ethiopian settings. Therefore, this study aimed to determine the incidence and predictors of LTFU among adults receiving ART in Guji zone, providing context-specific evidence to inform targeted interventions.

## Methods and materials

### Study area and study period

This research was carried out in Guji Zone, Oromia Region, Southern Ethiopia, in public health facilities that provide ART services. Over 1.6 million people live in Guji Zone, which also has 133 private clinics, 4 hospitals, 66 health centers, and 300 health posts. Merely 13 health centers and 2 hospitals provided comprehensive anti-retroviral prevention, care, and treatment services out of all these medical facilities [27, 28]. As of June 2022, 5,579 patients were receiving ART at health facilities in Guji Zone, with 3,914 (70%) of these enrolled at Shakiso Health Center, Negele General Hospital, Adola General Hospital, and Adola Health Center [27, 28].

According to the annual zonal health report, 459 clients (8.3%) were lost to follow-up from ART during July 2021–June 2022, representing the highest LTFU rate among zones in the region for that year [27, 28]. This study was done from February 15 to March 14, 2023, and included all public health facilities in Guji Zone that provided Anti-retroviral therapy services at the time of data collection.

#### Study design and populations

An institution-based retrospective follow-up study design was used. The study included adults aged ≥ 15 years who [1]: initiated ART between July 2018 and June 2022 [2], were registered in the ART register at the participating facility [9], had documentation of ART initiation. Patients were excluded if their medical records were missing completely, ART initiation or termination dates were not documented, or their final outcome (e.g., on care, dead, transferred out, LTFU) was unknown.

### Sample size determination and sampling technique

#### Sample size determination

The desired sample size was estimated using the power Cox command. Sample size was calculated using STATA Version 14 (power cox command) based on parameters from a previous study in Hadiya zone [29]: α = 0.05 (two-sided), power = 80%, and an LTFU incidence rate of 10.5 per 100 person-years. The calculation was performed for each significant predictor from that study using its adjusted hazard ratio. The predictor requiring the largest sample size was baseline CD4 count < 200 cells/mm^3^ with an adjusted hazard ratio (AHR) of 2.40, yielding a required sample of 394. After adjusting for 10% potential incomplete records, the final sample size was 434.

#### Sampling technique

**Fig. 1:**
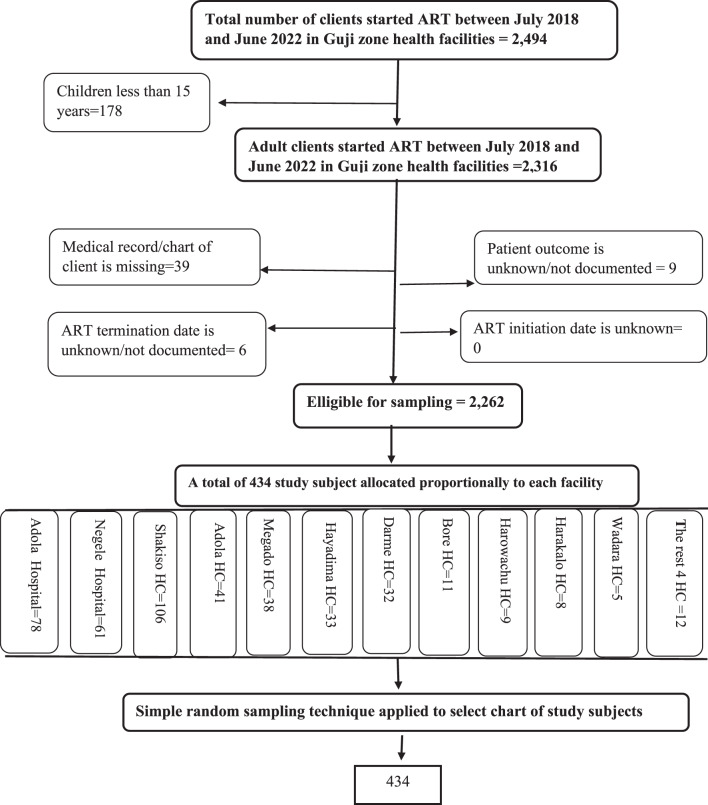
Schematic presentation of sampling procedure to assess the incidence of loss to follow up and its predictors among adults on anti-retro-viral therapy in Guji zone health facilities, southern Ethiopia, 2023

Each facility received a proportionate sample size based on the total number of adult clients who began ART at that institution between July 2018 and June 2022. From July 2018 to June 2022, Guji Zone health facilities enrolled 2,494 HIV-positive clients, including 2316 people aged ≥15 years who began using ART. After eliminating client charts for those who satisfied exclusion requirements, the unique ART numbers of eligible clients were sorted and used as a sampling frame for each facility. Finally, study participants were chosen using a simple random selection procedure with computer-generated random numbers (Fig. 1).

#### Data collection and its procedures

Data were collected using a structured abstraction checklist developed based on a review of relevant literature [6, 15, 21–23, 29–33] and adapted from Ethiopia’s Federal Ministry of Health ART intake and follow-up forms. Data were collected from an ART registration book, intake forms, patient follow-up charts, electronic medical records (EMR), medical history, and screening sheets used by Ethiopia’s Federal Ministry of Health. Initially, an electronic medical record and an ART register were utilized to determine those who were eligible for the study. Then, using the unique ART number from an electronic medical record or the ART register, additional patient data that was unavailable on the EMR or registration was obtained from patient follow-up records and other data sources. Permission was obtained from the Guji Zone Health Bureau, respective woreda health offices, and each health facility. Ten data collectors with qualifications in nursing, health informatics, or public health and experience in ART services were recruited and trained. A public health specialist supervised the data collection process.

### Study variables

#### Dependent variable

Incidence of loss to follow-up.

#### Independent variables

**Sociodemographic and economic factors**

Sex, occupation, age, religion, educational status, ethnicity, place of residence (urban/rural), housing condition (Homeless or shelter, Rented home, owner’s house), distance from the facility, marital status, and a number of children.

**Clinical and treatment factors**

The entry point of the client or model of HIV diagnosis (PITC model, ICT model, VCT model)

Time of ART initiation after diagnosis (early and late initiation of ART)

Baseline clinical variables (Presence of TB, Presence any OI, CD4, WHO clinical stage, weight, BMI, and functional status)

Clinical variables through follow-up (ART adherence, number of pills per day, type of ART regimen, ART regimen change, ART regimen failure, last CD4, last viral load, functional status, the incidence of TB and other OI after initiation, INH and CPT prophylactic therapy)

Drug side effects experienced (INH, cotrimoxazole, ART drug, and TB drug side effect)

Mental health illness

Multi month dispensing model (<3MMD, 3 MMD, 6 MMD)

Family and other support-related conditions

Presence of documented contact person/caregiver

Disclosure status

Serostatus of sexual partner

Partner engagement in care (If positive)

Having another HIV-positive family member

#### Operational definitions

**Event**: Loss to follow up

**Loss to follow-up:** The patient fails to visit ART clinics for more than 1 month since the last appointment date and has not been classified as “dead” or “transferring out” [6, 8, 15, 21, 33].

**Censored:** Patient doesn’t develop the event will be considered censored. This will include clients who are on follow-up, transfer out, and died.

**Rapid initiation:** initiation of antiretroviral therapy on the same day or within two weeks of confirmed HIV diagnosis according to the Ethiopian national ART guideline [6].

**Adherence:** Assessed on every clinical follow-up visit as good (>95%) missed ≤ 2 doses in 30 doses or ≤ 3 doses of 60 doses, fair (85–94%) missed 3–5 doses in 30 doses or 4–8 doses of 60 doses, and poor (<85%) missed ≥ 6 doses in 30 doses or ≥ 9 doses of 60 doses [6].

**Attrition:** Defined as discontinuation of ART for any reason. It includes death, loss to follow-up, stopping ARV medications while remaining in care [6, 8, 15, 21].

**Incomplete profile**: Defined as a scenario where the EMR, ART register, follow-up chart, or other paper-based forms in the patient’s medical record were unable to provide the data needed for this study.

**Working functional status:** “Capable of going out of home and routine activity including daily work” [6].

**Ambulatory functional status:** “Capable of doing self-care and going to the toilet unsupported” [6].

**Bedridden**: “Cannot go even to the toilet unsupported” [6].

**Transfer out:** is an official transfer out of the patient to another ART clinic after starting ART and recorded on a copy of the transfer out form, register, follow-up chart, or EMR.

**Advanced HIV Disease:** Patients were classified as having advanced HIV disease if they had a baseline CD4 count < 200 cells/mm^3^ or were in WHO clinical stage III or IV at ART initiation [6].

**Disclosure:** If a sexual partner or anyone knows the serostatus of the client at the workplace, school, family, and other community members [6].

#### Data quality management

To assure the quality of the data obtained and to address study variables, appropriate research was consulted when developing the data gathering instruments. The lead investigator conducted one day of training for data collectors and supervisors covering: study objectives, data collection procedures (what data to collect and from which sources), confidentiality protocols, and the roles and responsibilities of the research team. Furthermore, in order to assess the dependability of the data collection instrument, a pretest was conducted at the Abosto Health Center in Shashamane town utilizing 43 data abstraction sheets (10%) of the overall sample size. To guarantee the accuracy of the information gathered, supervision was done throughout the data collection phase. Prior to obtaining the completed forms from each data collector, the Principal investigator conducted a comprehensive review. In the interim, the patient charts and/or EMR data were chosen, and errors were immediately cross-checked for completeness.

#### Data entry, processing, and analysis

Following the completion of the data collection process, the data were coded, cleaned, and entered into Epi Data version 4.6.2. The dataset was subsequently transferred to STATA version 14.2 and SPSS version 25 for statistical analysis. Exploratory data analysis was conducted prior to the main analysis to identify incomplete entries, inconsistencies, and potential outliers, thereby ensuring overall data quality.

To prevent a substantial reduction in statistical power and to protect against the selection bias inherent in complete-case analysis, participants with missing data for key laboratory parameters specifically baseline CD4 cell counts and viral load measurements were not excluded from the cohort. Instead, a missing indicator approach was employed, whereby undocumented or undetermined laboratory results were retained as a distinct, independent analytical category (labeled as “Not determined”) within each respective variable. This strategy facilitated the preservation of the complete sample size across all bi-variable and multi-variable Cox proportional hazards regression models.

Tables were used to conduct descriptive statistics and display the results, giving a thorough rundown of the dataset. The date of ART beginning and the date of lost follow-up or censorship were taken into account when calculating the person’s time of observation.

The number of people who experienced loss to follow-up was divided by the total number of person years of observation on the study to determine the incidence rate of loss to follow-up. Six-month intervals were used to produce a life table that examined the cumulative survival time and the probability of loss to follow-up. Additionally, Kaplan-Meier survival curves were used to evaluate the cumulative chance of loss to follow-up following the initiation of ART. The overall survival status was compared across several variable categories using a log rank test, with a *p* value < 0.05 being statistically significant for the observed differences.

To find potential factors for the multivariable Cox regression, the bi-variable Cox regression was carried out. In order to find predictors of loss to follow-up, factors with a *p*-value less than 0.25 were added to the multivariable Cox regression following the bivariate analysis. The Schoenfeld residual test and the graphical approach were used to analyze the proportional hazard assumption. A variable was deemed to meet the assumption if its *p*-value on the Schoenfeld residual test was greater than 0.05. Multi variable Cox regression was then run by including selected candidate factors, and the adjusted hazard ratio (AHR), 95% confidence interval, and *p*-value of 0.05 were used to determine the statistical significance of the variables. The model’s overall goodness of fit was evaluated using the Cox-Snell residual test.

In order to rule out the interaction between the independent variables, multicollinearity was examined. Any multi-collinearity was found and addressed using a correlation coefficient more than 0.7 and a VIF threshold greater than 10.

## Results

### Baseline sociodemographic characteristics

This study reviewed the records of 434 HIV-infected adults who were newly enrolled to anti-retro-viral care and treatment at public health facilities in Guji zone between July 2018 and June 2022. Among the study subjects, 260 (60%) were female, and the age distribution was relatively evenly spread across the age categories, with the highest percentage in the 25–34 years category 175 (40.3%). The majority of the participants had either no formal education 159(36.4%) or primary education 169 (36.9%) (Table 1).

**Table 1:** Baseline sociodemographic characteristics of HIV positive adults on antiretroviral therapy in Guji zone, southern Ethiopia, 2023

Characteristics (*n* = 434)		Frequency	Percent (%)
Age categories	15–24 years	66	15.20
	25–34 years	175	40.30
	35–44 years	147	33.90
	≥45 years	46	10.60
Educational status	No formal education	159	36.40
	Primary (1–8)	160	36.90
	Secondary (9–12)	97	22.40
	Tertiary (diploma, degree and above)	18	4.10
Occupational Categories	Female sex worker	15	3.60
	Student	13	3
	Daily laborer and/or mobile worker	93	21.40
	House wife	98	22.60
	Employee (Govt/non govt)	41	9.40
	Farmer	91	21.20
	Self-Employee/private business	15	3.40
	Other	66	15.40
Religion	Orthodox	179	41.20
	Muslim	37	8.50
	Protestant	206	47.50
	Catholic	4	0.90
	Wakefata	8	1.80
Marital Status	Never Married	74	17.10
	Married	245	56.40
	Divorced	63	14.50
	Widowed	52	12.00
Family size	Live alone	103	23.70
	Two persons in home	93	21.40
	≥3 persons in home	238	54.80
Residence setting	Urban	251	57.80
	Rural	183	42.20
Residence relative to health facility	Same woreda as health facility	328	75.60
	Different woreda	106	24.40
Documented phone number	Yes	294	67.70
	No	140	32.30

### Patients clinical and laboratory related characteristics

At baseline, the majority 307 (70.7%) of patients had WHO stage 1 and 2 at baseline, while 127 (29.3%) had WHO stage 3 and 4. Similarly, more than ninety percent 402(92.60%) of patients had WHO stage 1 and 2 on their most recent visit, while only 32 (7.4%) had WHO stage 3 and 4. In terms of functional status, the majority 333 (76.7%) of participants were classified as working at baseline, while 78 (18.0%) were ambulatory and 23 (5.3%) were bedridden. On their most recent visit, 400 (94%) of patients were working, 20 (4.6%) were ambulatory, and 6 (1.4%) were bedridden (Table 2).

**Table 2:** Clinical and laboratory characteristics of HIV positive adults on antiretroviral therapy in Guji zone, southern Ethiopia, 2023

Characteristics (*n* = 434)		Frequency	Percent (%)
WHO stage on most recent visit	WHO stage 1 and 2	402	92.60
	WHO stage 3 and 4	32	7.40
Functional status on baseline	Working	333	76.70
	Ambulatory	78	18.00
	Bedridden	23	5.30
Baseline CD4	<200cells/mm^3^	84	19.40
	(200–350) cells/mm^3^	66	15.20
	≥350 cells/mm^3^	166	38.20
	Not determined	118	27.20
Advanced HIV diseases	Yes	153	35.30
	No	281	64.70
Nutritional status	Overweight	30	6.90
	Normal weight	279	64.30
	Under weight	120	27.60
Functional status on a most recent visit	Working	408	94.00
	Ambulatory	20	4.60
	Bedridden	6	1.40
The most recent Viral Load	Not detectable (Viral load < 50 Copies/ml)	302	69.58
	Low viremia (50–1000] copies/ml	37	8.50
	High Viral load (>1000)copies/ml	9	2.10
	VL undetermined/No VL test done	86	19.80
Presence of TB at the time of ART initiation	Yes	75	17.78
	No	359	82.72
Presence of any OI at the time of ART initiation	Yes	273	62.90
	No	161	37.10
Presence of any non-communicable disease	No	388	89.40
	Yes	46	10.60

### Adherence and treatment-related characteristics

Among clients included in this study, 295 (68%) have been receiving care treatment at a health center, while 139 (32%) received treatment at a hospital. The majority of clients 297 (68.4%) initiated ART within the same day of diagnosis and 337 (78%) of clients had good adherence treatment regimen on their most recent follow up month (Table 3).

**Table 3:** Adherence and treatment related characteristics of HIV positive adults on antiretroviral therapy in Guji zone, southern Ethiopia, 2023

Characteristics		Frequency	Percent (%)
Period of ART initiation after diagnosis	Within the same day	297	68.40
	Within two weeks	83	19.10
	After two weeks	54	12.40
Received TB preventive therapy	Yes	262	60.40
	No	172	39.60
Received Cotrimoxazole	Yes	182	41.90
	No	252	58.10
Regimen on baseline	TDF +3TC+DTG (1j)	245	56.50
	TDF +3TC+EFV (1e)	188	43.30
	TDF +3TC+NVP (1f)	1	0.20
	Others	0	0.00
ART Regimen on the last visit	TDF +3TC+DTG (1j)	392	90.30
	TDF +3TC+EFV (1e)	37	8.50
	Others	5	1.20
History of treatment failure	Yes	14	3.20
	No	420	96.80
Adherence status on the last month/most recent visit	Good	337	78.00
	Fair	50	11.60
	Poor	36	8.30
	Not assessed/Not documented	9	2.10
History of being non Adherent(fair/ poor adherence)	Yes	192	45.00
	No	235	55.00
History of Missed appointment before the recent appointment	Yes	75	17.30
	No	359	82.70
Documented History of drug side effect	Yes	42	9.80
	No	388	90.20

### Disclosure and support-related factors

Among the 434 clients whose records were reviewed, 321 (74%) had a contact person or caregiver, and 272 (62.7%) clients disclosed their HIV status (Table 4).

**Table 4:** Disclosure and support related factors of HIV positive adults on antiretroviral therapy in Guji Zone, southern Ethiopia, 2023

Disclosure and support related variable		Frequency	Percent (%)
Disclosed his/her sore-status	Yes	272	62.70
	No	162	37.30
Had sexual partner(documented)	Yes	242	55.80
	No	192	44.20
Sexual partner get tested for HIV	Yes	184	76.70
	No	56	23.30
Partner test result	Positive	141	76.60
	Negative	43	23.40
Partner enrolled in HIV care(From those positive)	Yes	122	86.52
	No	19	13.48

### Incidence of lost to follow-up after initiation of ART

A total of 434 participants were followed for up to 54 months (July 2018–June 2022), contributing 10,852 person-months (904.35 person-years) of observation. The study subjects were followed for a minimum of three months and a maximum of 54 months. Over the 4.5-year follow-up period, 135 (31.1%) participants were lost to follow-up, 23 (5.3%) died, 17 (3.9%) transferred out, and 259 (59.7%) remained on care at the same facility.

**Fig. 2:**
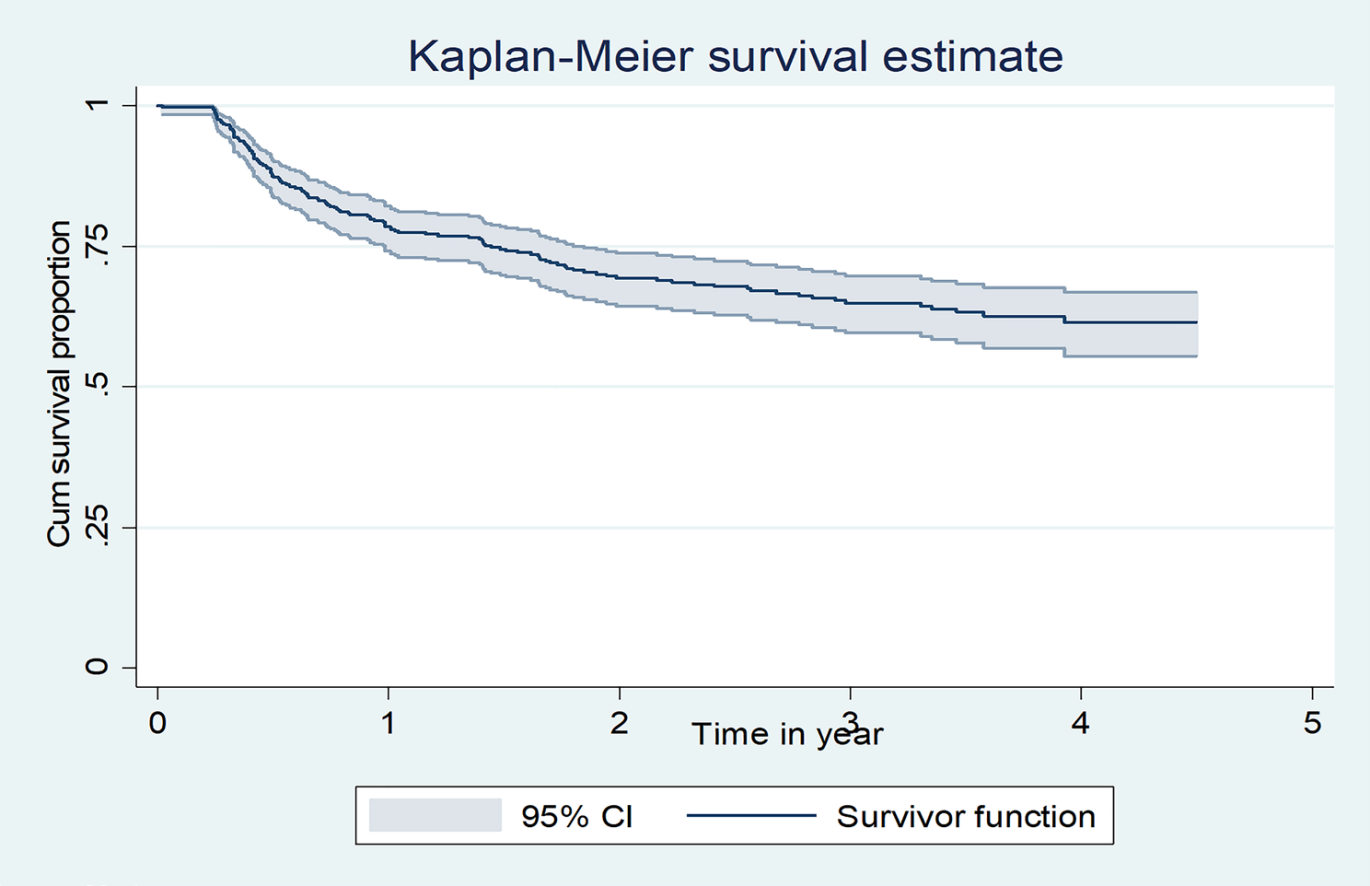
Overall Kaplan–Meier survival estimate of the loss to follow-up among adult patients attending the ART adults attending ART at health facilities in Guji zone, southern Ethiopia, 2023

The incidence of LTFU was found to be 14.93 per 100 person-years (95%CI: 14.88, 14.98), indicating that almost 15 study participants experienced LTFU for every 100 person-years of observation. The majority of LTFU incidents happened in the first one year. For instance, from all clients who experienced LTFU over 54 months, 52 (38.5%) had it within the first six months, while 89(66%) had it within the first 12 months. Additionally, this study demonstrated that the proportion of clients who survived to the end of the first, second, third, and fourth year was 78, 69, 65, and 62%, respectively (Table 5, Fig. 2).

**Table 5:** Survival status of HIV positive adults HIV positive adults on antiretroviral therapy in Guji zone, southern Ethiopia, 2023

Interval in month	# Entering Interval	# Withdrawing	Number Exposed to Risk	# Events (LTFU)	Proportion terminating	Probability density	Proportion of survival	Proportion of cumulative survival
0–6	434	17	426	52	0.12	0.020	0.88	0.88
6–12	365	35	348	37	0.11	0.016	0.89	0.78
12–18	293	27	279	14	0.05	0.007	0.95	0.75
18–24	252	39	232	16	0.07	0.009	0.93	0.69
24–30	197	18	188	4	0.02	0.002	0.98	0.68
30–36	175	26	162	7	0.04	0.005	0.96	0.65
36–42	142	40	122	3	0.02	0.003	0.98	0.63
42–48	99	47	75	2	0.03	0.003	0.97	0.62
48–54	50	50	25	0	0.00	0.000	1.00	0.62

**Fig. 3:**
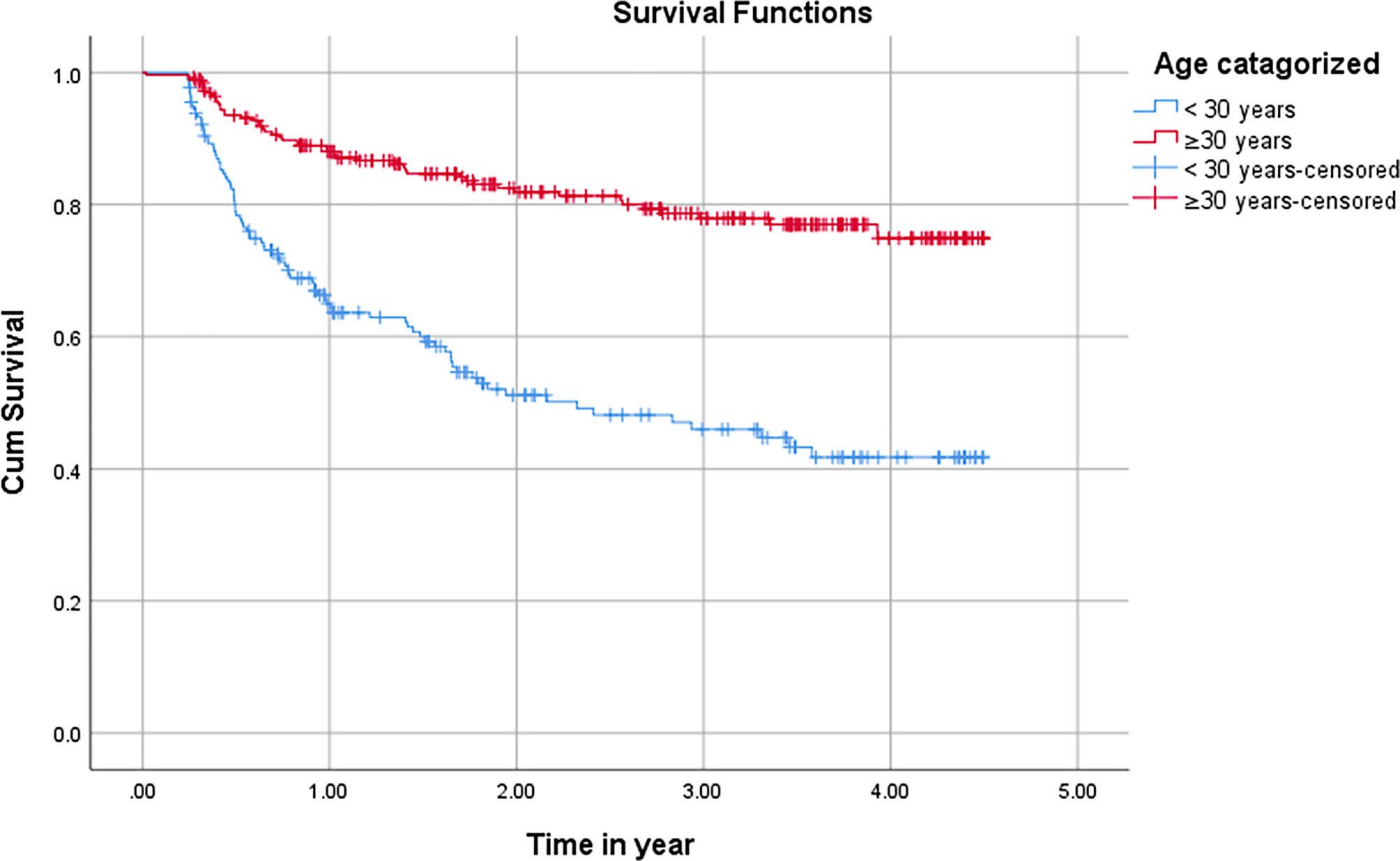
Comparison of survival status based on age categories of HIV-Positive adults attending ART at health facilities in Guji zone, southern Ethiopia, 2023

**Fig. 4:**
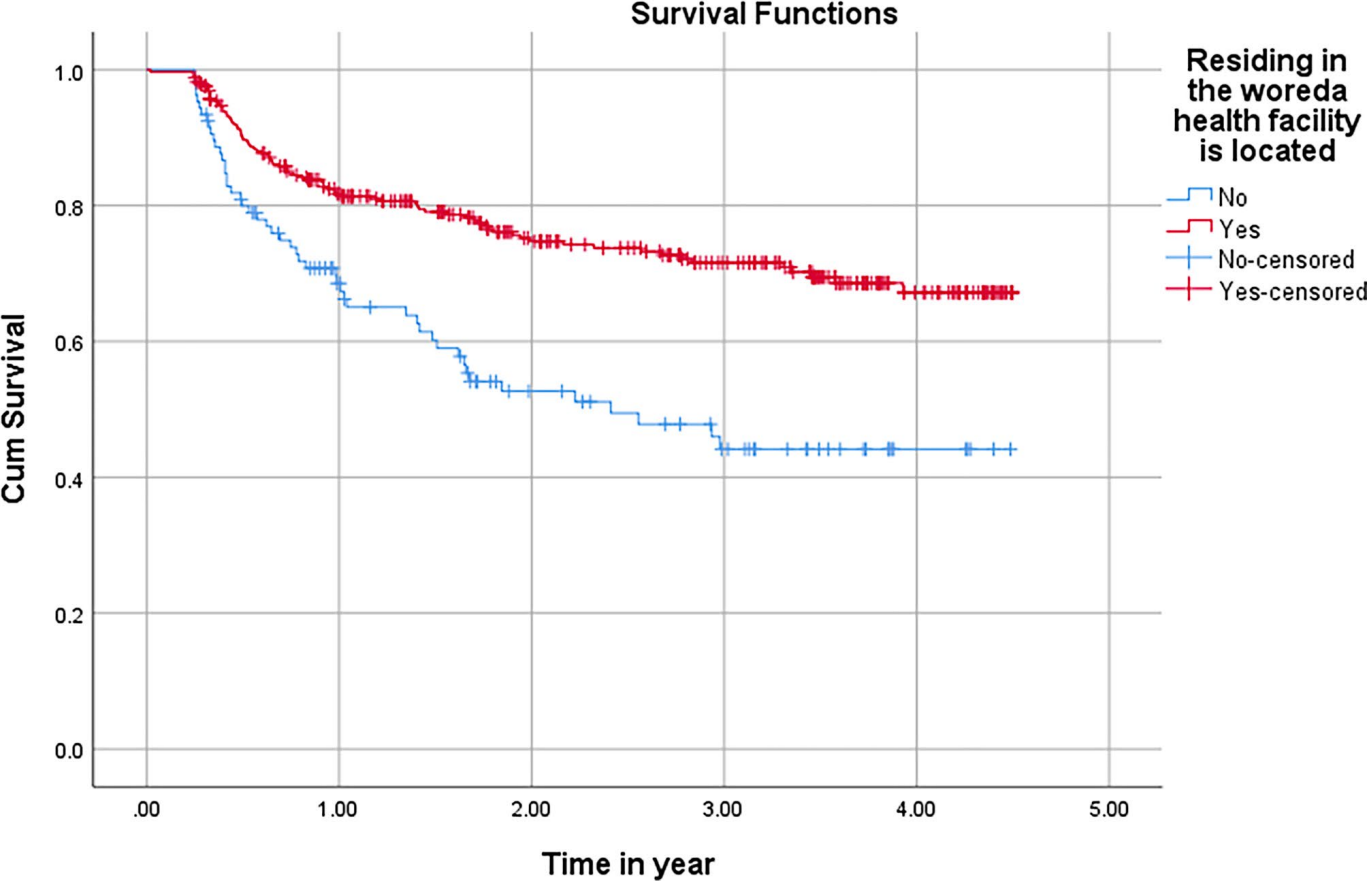
Comparison of survival status based on residence relative to health facility among HIV positive adults on antiretroviral therapy in Guji zone, southern Ethiopia, 2023

**Fig. 5:**
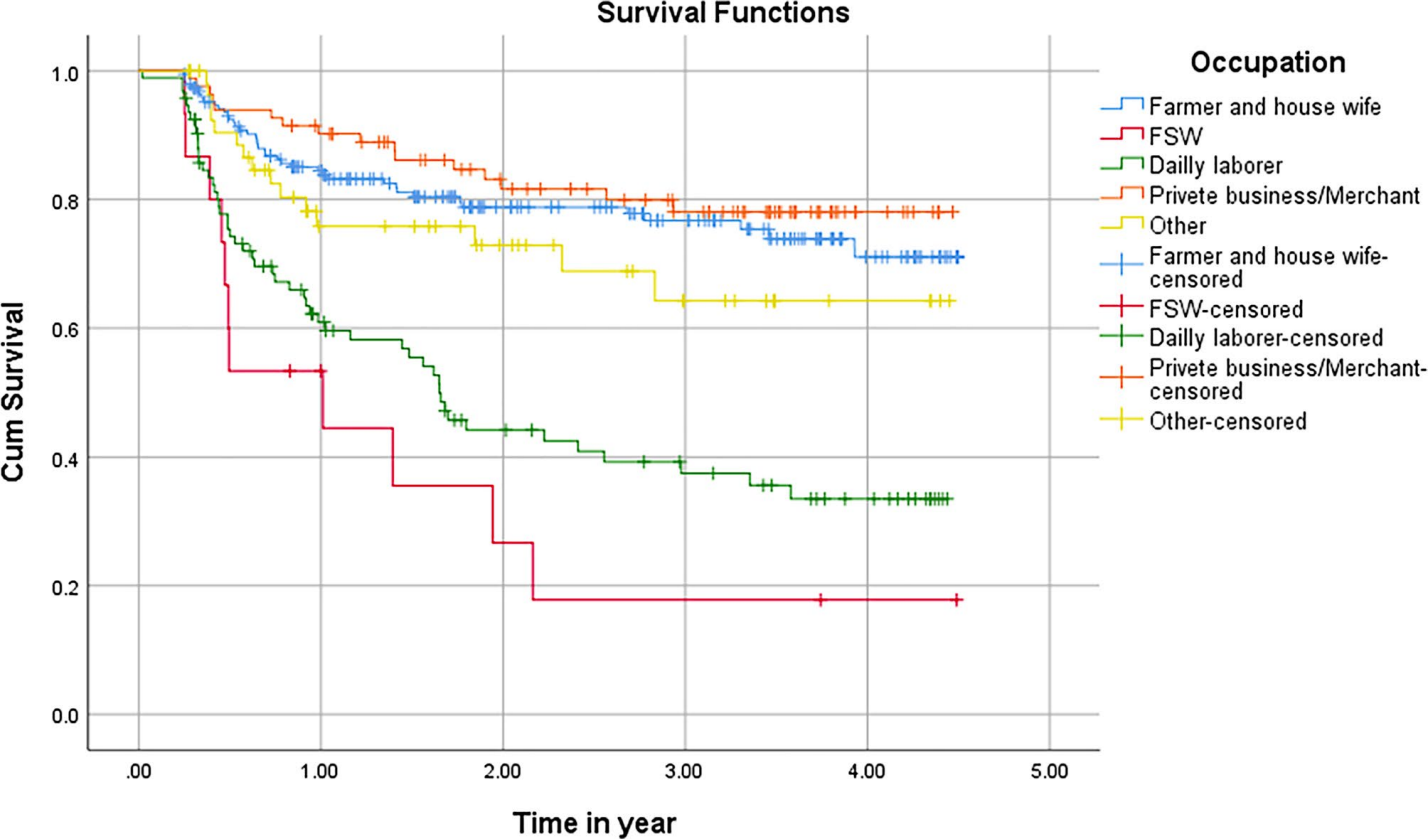
Comparison of survival status based on occupational category among HIV positive adults on antiretroviral therapy in Guji zone, southern Ethiopia, 2023

The log-rank test was used to determine if there were significant differences in survival status between different categories of variables. The results showed that there was statistically significant difference in the time loss to follow-up across different categories of sociodemographic variables, such as age (Chi-square value: 47.9, *p*-value: 0.001), residence in the same woreda as the health facility (Chi-square value: 21.3, *p*-value: 0.001), and occupation (Chi-square value: 66.6, *p*-value: 0.001). Comparison of survival function between categories of these variables shown on Figs. 3, 4, 5.

**Fig. 6:**
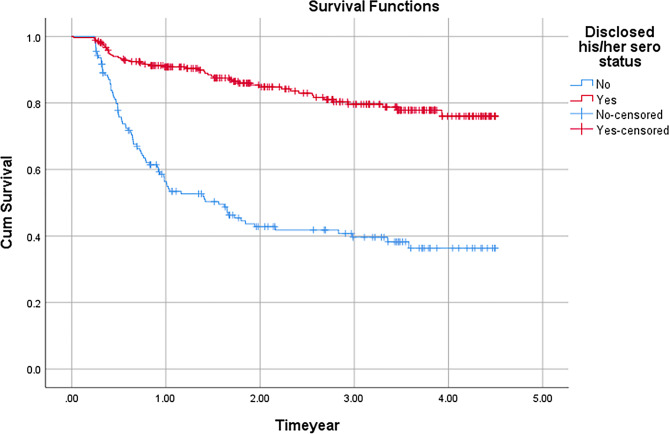
Comparison of survival function based on serostatus disclosure among HIV positive adults on antiretroviral therapy in Guji zone, southern Ethiopia, 2023

This study also took into account variables related to disclosure and family support. When conducting a log-rank test to compare hazard functions based on serostatus disclosure, the results of the log-rank test show a chi-square value of 78.88 with 1 degree of freedom and a *p*-value of 0.0001. This suggests that there was a significant difference in survival times between individuals who disclosed their serostatus and those who did not. As shown in the survival function graph below individuals who disclosed their serostatus to their sexual partners and/or other family members were less likely to discontinue their follow-up, in contrast to those who did not disclose their serostatus (Fig. 6).

### Predictors of loss to follow-up

Both bivariable and multivariable Cox regression analyses were used in the study to evaluate the predictors of loss to follow-up. The global Schoenfeld residual test (*p* = 0.9938) confirmed that the proportional hazards assumption was met. The multivariable Cox regression analysis identified several significant predictors of LTFU.

Participants who were younger than 30 years old had around 2.4 times higher risk of LTFU compared to those who were 30 years or older (AHR = 2.40, 95% CI: 1.58, 3.64). Similarly, male participants had 1.85 times higher risk of LTFU compared to female participants (AHR = 1.85, 95% CI: 1.27, 2.69). Participants who had no formal education had around two times higher risk of LTFU as compared to those who had at least primary level education (AHR = 2.20, 95% CI: 1.52, 3.20). On the other hand, commercial sex workers (FSWs) and daily laborers had a higher risk of LTFU compared to farmers and housewives (AHR = 3.26, 95% CI: 1.39, 7.65 and AHR = 1.91, 95% CI: 1.16, 3.16, respectively).

As shown in Table 6 below, individuals who belonged to the same woreda as the health facility had a 45% lower risk of LTFU compared to those who did not (AHR = 0.55, 95% CI: 0.38, 0.82). Additionally, clients with documented phone numbers on the record also had a lower risk of LTFU (AHR = 0.46, 95% CI: 0.31, 0.67) (Table 6).

**Table 6:** Cox regression analysis for predictors of lost to follow up among HIV positive adults on antiretroviral therapy in Guji zone, southern Ethiopia, 2023

		Survival status				
		Censored	Event (LTFU)	CHR (95% CI)	AHR (95% CI)	*p*-Value
		Count (%)	Count %			
Age	<30 years	93(52.00)	86(48.00)	3.24(2.29–4.60)	2.40(1.58–3.64)	0.001*
	≥30 years	206(80.80)	49(19.2)	1	1	
Sex	Male	104 (59.67)	70(40.23)	1.89(1.30–2.56)	1.85(1.27–2.69)	0.001*
	Female	195(75%)	65(25%)	1	1	
Marital	Never Married	32(43.2%)	42(56.8)	1	1	
	Married	188(76.73)	57(23.2)	0.31(0.20–0.46)	0.66(0.38–1.52)	0.145
	Divorced	40(63.5)	23(36.5)	0.53(0.32–0.88)	0.71(0.39–1.28)	0.256
	Widowed	39(75)	13(25)	0.35(0.19–0.65)	0.84(0.39–1.83)	0.662
Educational status	Had No formal education	92(57.90)	67(42.10)	2.08(1.48–2.92)	2.20(1.52–3.2)	0.001*
	Had at least primary education	207(75.27)	68(24.73)	1	1	
occupation	Farmer and housewife	148(78.3)	41(21.70)	1	1	
	FSW	4(26.7)	11(73.3)	5.13(2.63–10.00)	3.26(1.39–7.65)	0.007*
	Daily laborer	41(44.1)	52(55.9)	3.30(2.19–4.97)	1.91(1.16–3.16)	0.011*
	Private business/Merchant	66(80.50)	16(19.50)	0.77(0.43–1.37)	1.27(0.68–2.37)	0.453
	Others	40(72.7)	15(27.30)	1.40(0.78–2.53)	1.03(0.54–1.98)	0.923
Family size	Live alone	68(66.02)	35(33.98)	1	1	
	Two persons in a home	61(65.60)	32(34.40)	0.96(0.59–1.55)	0.90(0.54–1.49)	0.381
	≥3 persons in home	170(71.43)	68(28.57)	0.76(0.51–1.15)	0.80(0.52–1.24)	0.921
Residence Setting	Urban	172 (68.53)	79(31.47)	1	1	
	Rural	127(69.40)	56(30.60)	0.94(0.67–1.33)	0.92(0.62–1.35)	0.668
Residence relative to health facility	Same woreda as health facility	243(74.10)	85(25.90)	0.46(0.32–0.65)	0.55(0.38–0.82)	0.003*
	Different woreda	56(52.83)	50(47.16)	1	1	
Documented phone number	Yes	234(79.60)	60(20.40)	0.30(0.22–0.43)	0.46(0.31–0.67)	0.001*
	No	65(46.43)	75(53.57)	1	1	

Note: * indicates significant variable at α = 0.05

## Discussion

This study found that the overall incidence rate of loss to follow-up (LTFU) among adults receiving antiretroviral therapy (ART) was 14.93 per 100 person-years (PY). This finding is consistent with a similar study conducted in Bichena Health Center, Northwest Ethiopia, which reported an incidence rate of 13.45 per 100 PY [21]. However, the incidence rate of LTFU in this study was much higher than that reported in the majority of similar studies previously conducted in various parts of Ethiopia in which the incidence rates ranged from 3.5 to 12.26 per 100 PY [18, 21–25, 29, 31, 32, 34–36]. Besides, the incidence rate observed in this study is higher than the incidence rate observed in studies conducted in some other African countries [37–40]

Several factors may explain the higher LTFU incidence in this study compared to others.

These include transportation barriers, inadequate infrastructure, job insecurity among daily laborers requiring frequent movement, poor healthcare-seeking behavior, limited community-level patient education, and absence of community ART refill programs in the study area.

These factors might have directly or indirectly contributed to a higher likelihood of patients being lost to follow-up, which may have ultimately impacted the effectiveness of the Anti-retro viral care and treatment program in the study area.

On the other hand, LTFU incidence rate in this study (14.93/100PY) was lower when compared to incidence rate 26.6/100PY, 33.48LTFU/1000-person month and 46/100PY observed on study conducted at Karamara general hospital, Jigjiga town [33], Bunia, Democratic Republic of Congo respectively [30], and Liberia respectively [41]. This variation might be due to the difference in the study’s scope, as this study encompassed health centers at the district level, rather than solely focusing on a hospital. This could have resulted in a lower incidence of LTFU in this study, as these health centers were closer to patients’ residences. Furthermore, there may have been differences in the retention strategies employed and effectiveness of patient tracking system.

This study found that from the study subject entering each interval, the proportion of individuals exiting the study due to loss to follow-up (LTFU) decreased as the time interval increased. This suggests that the survival probability increased as the follow-up time increased. For example, the proportion of individuals lost to follow-up in the first six-month interval was 0.12, while in the second, third, and fifth six-month intervals, the proportions were 0.11, 0.05, and 0.02 respectively. This was consistent with what was seen in other studies of this nature [23, 33]. Furthermore, the finding indicates that early interventions may be particularly important in reducing LTFU, as the proportion of clients lost to follow-up decreases substantially after the first six-month interval.

Regarding factors that predict LTFU this study identified a number of factors that could be targets for future interventions. The finding showed that participants who were younger than 30 years old had two times higher risk of LTFU compared to those who were 30 years or older. (This finding is consistent with previous studies that have shown that younger individuals are more likely to disengage from care [22–24, 32, 33, 42]. Younger patients may have a higher risk of LTFU due to factors such as social and economic instability, lack of social support, stigma, and poor adherence to treatment. Additionally, their developmental stage may affect their ability to remain engaged in care. To address this issue, healthcare providers can implement targeted interventions such as peer support groups, psycho-social support, and educational programs.

Male participants in this cohort were significantly at a higher risk of discontinuing ART treatment as compared to their female counterparts. This is similar to other studies conducted previously [13, 39, 43–46]. The observed difference might be due to factors such as adherence to traditional masculine norms, engagement in risky behaviors or substance use, and social, cultural, or behavioral differences between male and females.

In congruent to other studies conducted previously [44, 45], being female sex workers significantly associated with increased risk of lost to follow up. This can be attributed to various factors, including stigma, privacy concerns, economic constraints, mobility nature of their work and health-seeking behavior. FSWs often face social stigma, which may discourage them from seeking healthcare services consistently.

Daily labor was also associated with increased LTFU risk, consistent with previous Ethiopian studies [24, 42]. Daily laborers often face economic constraints, time limitations, and job-related mobility that interfere with consistent clinic attendance. They may also face privacy issues, mobility challenges, and lack of awareness about the importance of ART adherence.

Individuals who lived in the same woreda as the health facility had a lower risk of LTFU suggesting that proximity to healthcare facilities plays a significant role in retention rates. This is congruent to study conducted previously in north west Ethiopia [46], Semarang City [43] and Kigali city [47].

### Limitations of the study

This study has certain limitations that should be considered when interpreting the findings. First, because the study relied on a retrospective review of routinely collected medical records, several variables were incompletely documented or missing in patient charts. In particular, a considerable proportion of participants lacked baseline CD4 cell counts, viral load measurements, and documented adherence status. These gaps were primarily driven by systemic and logistical challenges common to resource-limited health facilities, such as routine paper-chart under documentation, temporary laboratory reagent stock-outs, and limited institutional diagnostic capacity during specific periods of the cohort timeline.

Second, some patients categorized as lost to follow-up may have self-transferred to other health facilities or died without proper documentation, potentially leading to overestimation of the incidence of loss to follow-up. In addition,this study used a programmatic definition of loss to follow-up based on routine facility-level classification, where a patient is considered LTFU if they miss a scheduled ART appointment for more than 30 days and are not documented as transferred out or deceased. This definition is consistently used in Ethiopian public health facility recording systems, including ART registers, EMR, and follow-up charts. However, many studies use a 90-day definition, which may limit comparability with previous studies and potentially overestimate the incidence of loss to follow-up. Therefore, this limitation should be considered when interpreting and using the Finding of this Study.

## Conclusion

The results of the study showed that there was a significant incidence of follow-up loss among adult HIV-positive patients. A higher chance of loss to follow-up was substantially associated with male gender, age under 30, lack of formal education, working in commercial sex, and everyday labor. On the other hand, having a registered phone number and residing in the woredas where the medical facility is situated served as protective factors, lowering the possibility of losing follow-up. Reducing the loss of follow-up from care may be addressed through strengthening community-based care, enhancing professional capacity through patient engagement training, and offering counseling for ART users.

## Data Availability

Data supporting the findings of this study was obtained under applicable ethical and confidentiality guidelines. Due to privacy restrictions and proprietary information, the data of individual patients are not publicly available and not permitted to be provided to other bodies. However, the datasets generated and analyzed during this study are available from the corresponding author, Dereje Girma, at “derejegirma617@gmail.com” upon reasonable request. Interested researchers may contact corresponding author for potential access, subject to institutional and ethical approvals.

## Abbreviations

AIDS: Acquired Immune Deficiency Syndrome
AHR: Adjusted Hazard Ratio
ART: Anti-retroviral Therapy
BMI: Body Mass Index
CD4: Cluster of Differentiation 4
CI: Confidence Interval
CPT: Cotrimoxazole Prophylaxis Therapy
HAART: Highly Active Anti Retro-viral Therapy
HIV: Human Immunodeficiency Virus
INH: Isoniazid
IPT: Isoniazid Preventive Therapy
LTFU: Loss to Follow Up
MHI: Mental Health Illness
OI: Opportunistic Infection
PEPFAR: President’s Emergency Plan Fund for AIDS Relief
PLWHA: People Living With HIV/AIDS
PY: Pearson Year
SPSS: Statistical Packages for Social Science
SHR: Sub Hazard Ratio
SSA: Sub Saharan Africa
UNAIDS: United Nations Program on HIV/AIDS
WHO: World Health Organization
